# Supplementary material for: Practical Hamiltonian learning with unitary dynamics and Gibbs states
Source: Nat Commun. 2024 Jan 8;15:312. doi: 10.1038/s41467-023-44008-1 (PMC10774402; doi:10.1038/s41467-023-44008-1)
Supplement: Supplementary file 1 — Supplementary Information [file 41467_2023_44008_MOESM1_ESM.pdf]

Supplementary Notes: Practical Hamiltonian Learning with Unitary Dynamics and Gibbs States

Andi Gu,[1, 2, 3, *] Lukasz Cincio,[3] and Patrick J. Coles[3, 4]

[1] *Department of Physics, University of California, Berkeley, Berkeley, CA 94720, USA*
[2] *Harvard Quantum Initiative, Harvard University, Cambridge, MA 02138, USA*
[3] *Theoretical Division, Los Alamos National Laboratory, Los Alamos, NM 87545, USA*
[4] *Normal Computing Corporation, New York, New York, USA*

(Dated: November 1, 2023)

Supplementary Note 1 – Chebyshev Regression

To fit a black-box function f where we can query $f(x)$ for x within some window $[A,B]$, we are often interested in approximating f with a polynomial of degree L. This is known as polynomial interpolation. When we have complete freedom in choosing the location of the points $x \in [A,B]$, a popular method is Chebyshev interpolation. There are a number of favorable properties associated with this method. Importantly, it achieves close to an optimal approximation error on $[A,B]$. That is, if $\tilde{f}$ is the degree-L polynomial resulting from Chebyshev interpolation, $\max_{x\in[A,B]} \left| f(x) - \tilde{f}(x) \right|$ is close to the minimal possible value among all polynomials of degree L [1, 2].

Chebyshev interpolation takes its name from its extensive use of a class of polynomials known as Chebyshev polynomials. This class of polynomials is unique in that for $z \in [-1,1]$, they can be written:

$$T_n(z) = \cos(n \arccos z) \tag{1}$$

Although this is not manifestly in polynomial form, one can show [1] that an equivalent definition is

$$T_n(z) = \sum_{r=0}^{\lfloor n/2 \rfloor} (-1)^r \binom{n}{2r} z^{n-2r}(1-z^2)^r. \tag{2}$$

This makes clear that the degree of T_n is n. Below, we list a number of useful properties of Chebyshev polynomials.

Lemma 1. The roots of the nth Chebyshev polynomial are

$$z_m = -\cos\left(\frac{2m-1}{2n}\pi\right), \quad m = 1, \ldots, n \tag{3}$$

Proof. This follows from a simple substitution of Equation (3) into Equation (1).

Lemma 2. If $\{z_m \mid m = 1, \ldots, n\}$ are the roots of T_n, then for any $i, j \le n$:

$$\sum_{m=1}^{n} T_i(z_m) T_j(z_m) = \begin{cases} 0 & \text{if } i \neq j \\ \frac{n}{2} & \text{if } i = j \neq 0 \\ n & \text{if } i = j = 0 \end{cases} \tag{4}$$

In the general case, if either $i > n$ or $j > n$:

$$\sum_{m=1}^{n} T_i(z_m) T_j(z_m) = \begin{cases} \frac{n}{2} & \text{if either } i+j \mid 2n \text{ or } i-j \mid 2n \\ n & \text{if } (i+j) \mid 2n \text{ and } i-j \mid 2n \\ 0 & \text{otherwise} \end{cases} \tag{5}$$

Proof. See Section 4.6 of Mason and Handscomb [1].

* andigu@g.harvard.edu

With these preliminaries, we now describe Chebyshev interpolation. After fixing a degree $L-1$ for the interpolating polynomial, we evaluate f at the roots of $T_L(z)$. This assumes the window in which we are approximating f is $[-1,1]$, however, the generalization to arbitrary windows is straightforward. This results in a dataset $\mathcal{D} = \{(z_i, y_i) \mid i = 1, \ldots, L\}$ where z_i are the roots of $T_L(z)$ and $y_i = f(z_i)$. The functional form with which we interpolate $\{y_i \mid i = 1, \ldots, L\}$ is simply a linear combination of the first L Chebyshev polynomials:

$$\tilde{f}(z; \{b_i\}) = \sum_{\ell=0}^{L-1} b_\ell T_\ell(z), \tag{6}$$

where the coefficients $b_\ell \in \mathbb{R}$ are parameters that we fit to the data.

Theorem 1 (Chebyshev interpolation). The coefficients $\{b_i\}$ such that $\tilde{f}(z; \{b_i\})$ perfectly fits our dataset are:

$$b_\ell = \begin{cases} \frac{1}{L}\sum_{i=1}^{L} y_i & \text{for } \ell = 0 \\ \frac{2}{L}\sum_{i=1}^{L} y_i T_\ell(z_i) & \text{for } \ell \neq 0 \end{cases} \tag{7}$$

Proof. We minimize the squared distance squared error between $\tilde{f}$ and the dataset. Then, since any set of L points can be perfectly fitted by a degree $L-1$ polynomial, the resulting coefficients will be the interpolation solution. We set $\sum_i \frac{\partial}{\partial b_\ell}\left(y_i - \tilde{f}(z_i; B)\right)^2 = 0$, which results in

$$\sum_{i=1}^{L} y_i T_\ell(z_i) = \sum_{m=0}^{L-1} b_m \sum_{i=1}^{L} T_\ell(z_i) T_m(z_i).$$

Applying Lemma 2, we find that b_0 satisfies

$$b_0 = \frac{1}{L}\sum_{i=1}^{L} y_i,$$

which simply says that the constant term in the fitted polynomial is the average over $\{y_i\}$, an intuitive result. For $\ell \neq 0$:

$$b_\ell = \frac{2}{L}\sum_{i=1}^{L} y_i T_\ell(z_i).$$

Supplementary Note 2 – Bounding Iterated Commutators

Definition 1 (Types of tuples). We introduce some nomenclature that abbreviates much of our later proofs. First, let Θ be a tuple of the Hamiltonian parameters $(\theta_1, \ldots, \theta_r)$. Also, we introduce multi-index sets $\alpha \in (\mathbb{Z}_{\geq 0})^r$, which are tuples of nonnegative integers $(\alpha_1, \alpha_2, \ldots, \alpha_r)$. We define:

$$|\alpha| = \sum_i \alpha_i, \quad \Theta^\alpha = \prod_i \theta_i^{\alpha_i}. \tag{8}$$

We call $|\alpha|$ the size of α. Finally, we define "term tuples" $S \in \{1, \ldots, r\}^m$ of size m to be ordered tuples $(s_1, \ldots, s_m)$. Each entry in S is meant to point at a term in the Hamiltonian.

There is a correspondence between size m multi-index sets and term tuples. A term tuple S with size m maps to α as follows: $(\alpha(S))_i = |\{j \mid S_j = i, j = 1, \ldots, m\}|$. This mapping essentially counts the number of occurrences of i in S. Graphically, this is represented in Figure 1. This mapping is surjective, in the sense that any multi-index set α with size m can be written as $\alpha(S)$ for some S with size m. However, it is not injective, since multiple term tuples can map to the same multi-index set.

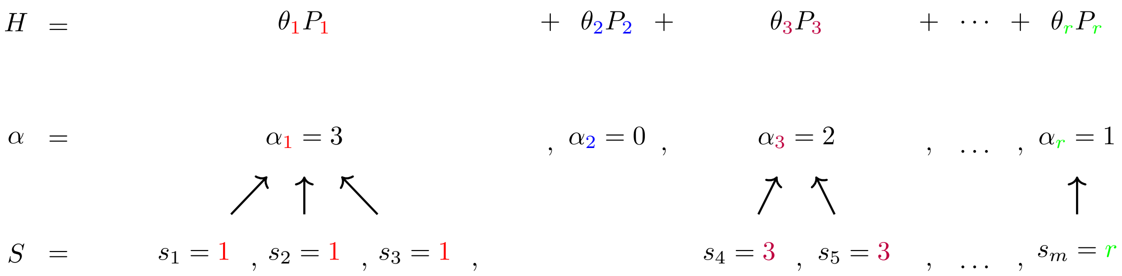

Supplementary Figure 1: The correspondence of $s_1, \dots, s_m$ with $\alpha_1, \dots, \alpha_n$. As the colors indicate, the value of each s_i indicate a term in the Hamiltonian (e.g., $s_i = \mathbf{3}$ refers to the $\boldsymbol{\theta_3 P_3}$ term).

Lemma 3. The iterated commutator $[H^m P]$ can be expanded as:

$$[H^m P] = \sum_{S \in \{1,\dots,r\}^m} \Theta^{\alpha(S)} [P_{s_1}, [P_{s_2}, \dots, [P_{s_m}, P]]]. \tag{9}$$

We emphasize that Equation (9) is nothing more than a polynomial expansion, in the Hamiltonian coefficients, of the iterated commutator.

Proof. We inductively arrive at the following formula for the iterated commutator $[H^m, P]$.

$$[H^m P] = \sum_{s_1=1}^{r} \sum_{s_2=1}^{r} \underset{m \text{ times}}{\dots} \sum_{s_m=1}^{n} \theta_{s_1}\theta_{s_2}\dots\theta_{s_m} [P_{s_1}, [P_{s_2}, \dots, [P_{s_m}, P]]]. \tag{10}$$

This follows simply from the linearity of the commutator (i.e., $[A + B, C] = [A, C] + [B, C]$):

$$\left[H^{m+1} P\right] = [H, [H^m P]] \tag{11a}$$

$$= \left[H, \sum_{s_1=1}^{r} \sum_{s_2=1}^{r} \dots \sum_{s_m=1}^{r} \theta_{s_1}\theta_{s_2}\dots\theta_{s_m} [P_{s_1}, [P_{s_2}, \dots, [P_{s_m}, P]]]\right] \tag{11b}$$

$$= \sum_{s_{m+1}=1}^{r} \sum_{s_1=1}^{r} \dots \sum_{s_m=1}^{r} \theta_{s_1}\dots\theta_{s_m}\theta_{s_{m+1}} \left[P_{s_{m+1}}, [P_{s_1}, \dots, [P_{s_m}, P]]\right], \tag{11c}$$

where Equation (11b) follows from the induction hypothesis and Equation (11c) follows from the linearity of the commutator. This gives the desired result, up to a relabeling of the indices. These tuples are members of $(\mathbb{Z}_n)^m$, so we rewrite Equation (9) as:

$$[H^m P] = \sum_{S \in \{1,\dots,r\}^m} \theta_{s_1}\dots\theta_{s_m} [P_{s_1}, [P_{s_2}, \dots, [P_{s_m}, P]]]$$

$$= \sum_{S \in \{1,\dots,r\}^m} \Theta^{\alpha(S)} [P_{s_1}, [P_{s_2}, \dots, [P_{s_m}, P]]]$$

Without any structural assumptions about the Hamiltonian H, the form of this expansion is not useful. However, by applying our assumption that the Hamiltonian is sparsely interacting, we can find useful bounds using the above expansion.

Definition 2 (Support tree). Let P be any Pauli operator such that there is some P_i where $\operatorname{supp} P \subseteq \operatorname{supp} P_i$ (for the remainder of this work, wherever we write P, it will always satisfy this assumption). For this Pauli, we can define the support tree induced by P, which we call $\mathcal{T}_P$ (see Figure 2). This tree is defined as follows: the children of any node P_i is

$$\text{children}(P_i) = \{P_j \mid \text{supp}(P_i) \cap \text{supp}(P_j) \neq \varnothing\}.$$

The root of $\mathcal{T}_P^{(m)}$ is P.

Example 2.1. The following is a support tree induced by $P = \sigma_x^{(2)}$ on the 9-qubit TFIM shown in Figure 2 of the main text.

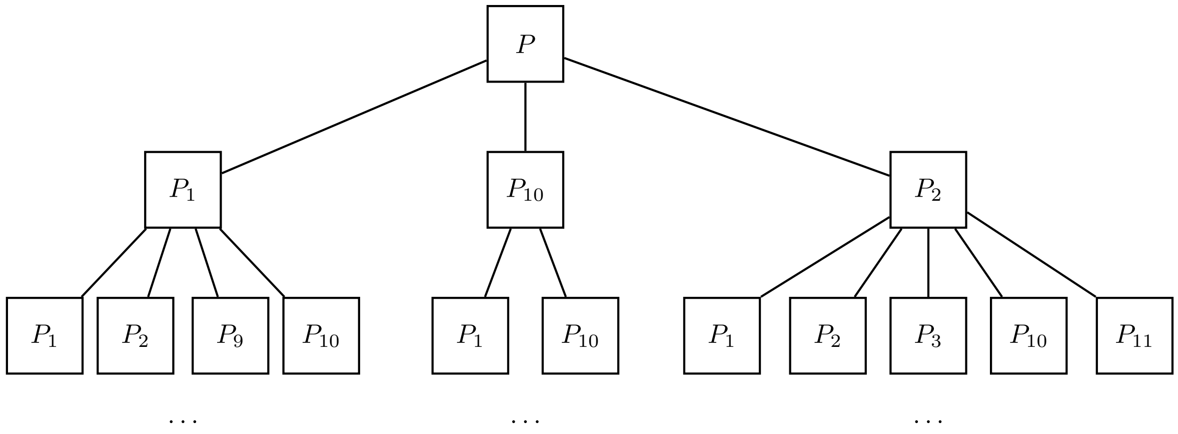

Supplementary Figure 2: The support tree $\mathcal{T}_P$ for a 9-qubit TFIM system, induced by some Pauli P that acts on qubit 2 (e.g., $P = \sigma_x^{(2)}$). For illustration purposes, we truncate the tree at a depth of 2. Each node has at most $\mathscr{D}+1$ children. The structure of this tree is entirely dependent on the interaction graph.

Theorem 2 (Counting labeled subtrees). Let $\mathcal{T}$ be an infinite rooted d-regular tree (i.e., every node has d children). There are exactly

$$a_m = \prod_{n=1}^{m-1} (n(d-1)+1) \leq (d-1)^{m-1} m! \tag{12}$$

labelled rooted subtrees in $\mathcal{T}$, of size m and labels $\{1, \ldots, m\}$, such that the label of any given node is less than that of all of its descendants.

Proof. Let a_m be the number of labelled rooted subtrees of size m that satisfy the requirements in the above theorem. If we neglect the labeling, so that any subtrees are identical if they are structurally identical, then a_m satisfies the following recurrence:

$$a_m = \sum_{\substack{0 \leq k_1, \ldots, k_d \leq d \\ \sum k_j = m-1}} \prod_{i=1}^{d} a_{k_i} \tag{13}$$

Each summand represents a configuration in which the root node has a tree of size k_1 on its first child, size k_2 on its second child, and so on. We now need to adjust this recurrence to account for labeling.

For some configuration $(k_1, k_2, \ldots, k_d)$, observe that we can combine the configurations as follows. There are m slots that need to be labeled. The first must be the root. Then, we can place the labels from the first subtree in any of $\binom{m-1}{k_1}$ positions (while maintaining relative ordering within the subtree, which preserves the property that the label of any given node is less than that of all its descendants). Next, we can place the labels from the second subtree in any of $\binom{m-1-k_1}{k_2}$ positions, and so on. In total, there are $\frac{(m-1)!}{k_1!(m-1-k_1)!} \frac{(m-1-k_1)!}{(m-1-k_1-k_2)!k_2!} \cdots = \frac{(m-1)!}{k_1! k_2! \ldots k_d!}$ ways of placing the labels. This is simply the multinomial coefficient $\binom{m-1}{k_1, \ldots, k_d}$. Therefore, the correct recurrence relation is:

$$a_m = \sum_{\substack{0 \leq k_1, \ldots, k_d \leq d \\ \sum k_j = m-1}} \frac{(m-1)!}{k_1! \ldots k_d!} \prod_{i=1}^{d} a_{k_i}. \tag{14}$$

This can be written in a more convenient form if we define a $b_m \equiv \frac{a_m}{m!}$, so that we have:

$$b_m = \frac{1}{m} \sum_{\substack{0 \leq k_1, \ldots, k_d \leq d \\ \sum k_j = m-1}} \prod_{i=1}^{d} b_{k_i}. \tag{15}$$

To obtain a closed form for b_m, we define the generating function $G(z) = b_0 + b_1 z + b_2 z^2 + \ldots = \sum_m b_m z^m$.

$$
\begin{aligned}
(G(z))^d &= \sum_{m=0}^{\infty} \left(\sum_{\substack{0 \le k_1, \ldots, k_d \le d \\ \sum k_j = m}} \prod_{i=1}^{d} b_{k_i} \right) z^m \\
&= \sum_{m=0}^{\infty} (m+1) b_{m+1} z^m \\
&= G'(z)
\end{aligned}
$$

It can be verified that

$$G(z) = \sqrt[-\alpha]{-\alpha(z - \alpha^{-1})} \tag{16}$$

for $\alpha \equiv d - 1$ satisfies this relation. Now, observe that $\left|\frac{\mathrm{d}^m G}{\mathrm{d}z^m}\right|_{z=0} = b_m m! = a_m$. We show by induction that

$$\frac{\mathrm{d}^m G}{\mathrm{d}z^m} = \frac{\prod_{n=1}^{m-1}(n\alpha + 1)}{(1 - \alpha z)^{1+1/\alpha}} \tag{17}$$

For the base case $m = 1$, the derivative is $\frac{1}{(1-\alpha z)^{1+1/\alpha}}$. Then, assuming the induction hypothesis,

$$\frac{\mathrm{d}^{m+1} G}{\mathrm{d}z^{m+1}} = \frac{\mathrm{d}}{\mathrm{d}z} \frac{\prod_{n=1}^{m-1}(n\alpha + 1)}{(1 - \alpha z)^{1+1/\alpha}} = \frac{\prod_{n=1}^{m}(n\alpha + 1)}{(1 - \alpha z)^{1+1/\alpha}}$$

This completes the induction. Finally, we see that:

$$a_m = \prod_{n=1}^{m-1}(n\alpha + 1) \le \alpha^{m-1} \prod_{n=1}^{m-1}(n+1) = (d-1)^{m-1} m! \tag{18}$$

Corollary 2.1. We define a non-vanishing tuple S of size m as a tuple where

$$[P_{s_1}, \ldots, [P_{s_m}, P]] \neq 0.$$

There are at most $\mathscr{D}^m (m+1)!$ non-vanishing tuples of size m.

Proof. We argue that non-vanishing tuples are in one-to-one correspondence with the subtrees of size $m+1$ described in the previous theorem. We do this by construction.

Algorithm 1 Finding all possible non-vanishing tuples of size m

```
procedure NonvanishingTuples(S, F, m)
    if m = 0 then
        return [P]
    else
        R ← []
        for v ∈ F do
            S[m] ← v
            F′ ← F                                   ▷ Copy F to F′
            Remove v from F′
            Add the children of v to F′
            R.extend(NonvanishingTuples(S, F′, m − 1))
        return R
S_0 ← empty list of size m + 1
S_0[m + 1] ← P
F_0 ← the children of P in the support tree of T_P
{S} ← NonvanishingTuples(S_0, F_0, m)
```

In the above, S represents a given non-vanishing tuple, and F represents a frontier of Paulis whose support intersects with the support of at least one Pauli in S. This algorithm builds non-vanishing tuples from inside-out, first selecting

all P_{s_m} such that $\operatorname{supp} P_{s_m} \cap \operatorname{supp} P \neq \varnothing$, then finds $P_{s_{m-1}}$ such that $\operatorname{supp} P_{s_{m-1}} \cap (\operatorname{supp} P_{s_m} \cup \operatorname{supp} P) \neq \varnothing$, and so on.

Now, we observe that this algorithm is constructing, structurally speaking, rooted subtrees of size $m+1$ in $\mathcal{T}_P$ (where the $+1$ is due to the root P). The recursion takes the current subtree, searches its 'frontier' (i.e., the set of nodes that is connected to at least one node in the current subtree) and adds one of these frontier nodes to the current subtree, repeating until the subtree is size $m+1$. However, the algorithm does not only output structures: there is an ordering to each structure depending on the order in which node is visited. A given subtree may be counted several times, with different ordering. This ordering corresponds to a labeling of the nodes in the subtree with $\{1, \ldots, m\}$, with the labels representing the order in which the nodes are visited. The constraint on the labeling is that no descendent of a node can be visited before its parent – this simply means the label of any node must be strictly less than that of its descendants. Therefore, Algorithm 1 counts all labelled subtrees of size m with the property that the label of any node is less than that of its descendants. Since $\mathcal{T}_P$ is at most $(\mathscr{D}+1)$-regular, by Theorem 2, there are at most $\mathscr{D}^m(m+1)!$ non-vanishing tuples.

Remark 2.1. It is not guaranteed that every tuple returned by Algorithm 1 will be nonvanishing, since it is possible, for example, that P_{s_1} commutes with $[P_{s_2}, \ldots, [P_{s_m}, P]]$. However, it is guaranteed that every non-vanishing tuple is contained in the set $\{S\}$ found by Algorithm 1.

Remark 2.2. Corollary 2.1 is a similar approach to [3]. However, they use the known result that the number of structurally unique subtrees with size m of a d-regular tree is $\frac{1}{(d-2)m+1}\binom{(d-1)m}{m}$ [4, 5]. This scales with $\sim (e \cdot d)^m$, and if we want to count all non-vanishing tuples, to allow for relabelling, we are forced to introduce a factor $m!$. This gives a bound on the number of non-vanishing tuples $\sim (e \cdot d)^m m!$. By being more careful with relabeling (since not all labelings are permitted), the counting in Theorem 2 is better by a factor $\mathcal{O}\left(\frac{e^m}{m}\right)$.

Lemma 4. For any single qubit observable P that satisfies $\|P\| \leq 1$, the spectral norm of the iterated commutator is bounded by

$$\|[H^m P]\| \leq (2\mathscr{D}\|\Theta\|_\infty)^m (m+1)! \tag{19}$$

where the ℓ_∞ norm denotes $\|\Theta\|_\infty = \max_i |\theta_i|$.

Proof. We refer to the expression for the iterated commutator in Equation (9):

$$[H^m P] = \sum_{S \in \{1,\ldots,r\}^m} \Theta^{\alpha(S)} [P_{s_1}, [P_{s_2}, \ldots, [P_{s_m}, P]]]$$

First, each commutator in this sum has norm at most 2^m, since $\|[A,B]\| \leq 2\|A\|\|B\|$ and all Paulis have spectral norm $\|P\| = 1$. Also,

$$\Theta^{\alpha(S)} = \prod_{i=1}^{n} \theta_i^{\alpha(S)_i} \leq \prod_{i=1}^{n} \|\Theta\|_\infty^{\alpha(S)_i} = \|\Theta\|_\infty^{|\alpha(S)|} = \|\Theta\|_\infty^m$$

Finally, by Corollary 2.1, there are at most $\mathscr{D}^m(m+1)!$ non-vanishing term tuples. Combining these together, we get that

$$\begin{aligned} \|[H^m P]\| &\leq \sum_{S \in \{1,\ldots,r\}^m} \Theta^{\alpha(S)} \|[P_{s_1}, [P_{s_2}, \ldots, [P_{s_m}, P]]]\| \\ &\leq \underbrace{\|\Theta\|_\infty^m}_{\text{From } \Theta^{\alpha(S)}} \quad \underbrace{2^m}_{\text{From each commutator}} \quad \underbrace{\mathscr{D}^m(m+1)!}_{\text{Counting non-vanishing } S} \\ &= (2\mathscr{D}\|\Theta\|_\infty)^m (m+1)! \end{aligned} \tag{20}$$

Theorem 3. For commuting Hamiltonians (i.e., every term P_i in the Hamiltonian commutes with every other term), when P is a single-qubit observable

$$\|[H^m P]\| \leq (2(\mathscr{D}+1)\|\Theta\|_\infty)^m \tag{21}$$

for all m.

Proof. We separate $H = H_1 + H_2$, where H_1 is composed of all terms in the Hamiltonian that have a support that overlaps with the support of P. We then inductively show Equation (21) by proving the strong statement

$$[H^m P] = [H_1^m P].$$

Assuming the induction hypothesis, we have:

$$\left[H^{m+1}P\right] = \left[H_1^{m+1}P\right] + [H_2, [H_1^m P]]$$

Using the identity $[A^m B] = \sum_{k=0}^{m}(-1)^k\binom{m}{k}A^{m-k}BA^k$:

$$= \left[H_1^{m+1}P\right] + \sum_{k=0}^{m}(-1)^k\binom{m}{k}\left(H_2 H_1^{m-k} P H_1^k - H_1^{m-k} P H_1^k H_2\right)$$

By commutativity of the Hamiltonian $[H_1, H_2] = 0$, so we can freely rearrange H_2 and H_1. That is, $H_2 H_1^{m-k} P H_1^k = H_1^{m-k} H_2 P H_1^k$ and similarly $H_1^{m-k} P H_1^k H_2 = H_1^{m-k} P H_2 H_1^k$. This gives:

$$= \left[H_1^{m+1}P\right] + \sum_{k=0}^{m}(-1)^k\binom{m}{k} H_1^{m-k}[H_2, P]H_1^k$$

By definition of H_2, $\operatorname{supp} H_2 \cap \operatorname{supp} P = \varnothing$, so they commute.

$$= \left[H_1^{m+1}P\right]$$

Since H_1 contains at most $\mathscr{D} + 1$ terms, by applying the triangle inequality for the matrix norm, we have $\|H_1\| \leq \|\Theta\|_\infty(\mathscr{D} + 1)$. Finally, applying $\|[A^m B]\| \leq 2^m \|A\|^m \|B\|$, we find $\|[H^m P]\| \leq (2(\mathscr{D} + 1)\|\Theta\|_\infty)^m$, as desired.

Notably, Equation (21) is smaller than the general bound in Equation (19) by a factor $\sim m!$. This can be attributed to the fact that when H is commuting, the support $[H^m P]$ never grows beyond a ring around P – more precisely, $\operatorname{supp}\,[H^m P] \subseteq \operatorname{supp} H_1$ for all m.

Supplementary Note 3 – Proof of Theorem 2

We first establish one preliminary: since we use Chebyshev regression, it is natural to first write the expectation in the form $\langle P(t)\rangle = \sum_{m=0}^{\infty} b_m T_m(2t/A - 1)$, where T_m is the mth Chebyshev polynomial. However, we need to find an expression relating b_m to the Taylor expansion coefficients – specifically, since we are interested in expressing c_1 as a function of $\{b_m\}$.

Lemma 5. If we have a polynomial of degree L represented in the Chebyshev basis

$$f(t) = \sum_{m=0}^{L} b_m T_m(2t/A - 1),$$

if we write the same polynomial with $f(t) = \sum_{m=0}^{L} c_m t^m$, we have

$$c_1 = -\frac{2}{A}\sum_{m=1}^{L}(-1)^m b_m m^2 \tag{22}$$

Proof. Since $c_1 \equiv f'(0)$:

$$\begin{aligned} c_1 &= \sum_{m=1}^{L} b_m \frac{\mathrm{d}}{\mathrm{d}t}\, T_m(2t/A - 1)\Big|_{t=0} \\ &= \sum_{m=1}^{L} \frac{2}{A} b_m T_m'(-1) \end{aligned}$$

We apply the formula $T_m'(-1) = (-1)^{m+1}m^2$ [1, Section 2.4.5].

$$= -\frac{2}{A}\sum_{m=1}^{L}(-1)^m b_m m^2.$$

Now, since it appears frequently in the following proofs, we will introduce a shorthand

$$\gamma \equiv 2\mathscr{D}\|\Theta\|_\infty = \tau^{-1} \tag{23}$$

to define a typical scale for the Hamiltonian coefficients.

Theorem 4 (Error bound). The estimator $\tilde{c}_1$ proposed in Algorithm 2 of the main text achieves an error:

$$\mathbb{E}\left[\frac{(c_1-\tilde{c}_1)^2}{\gamma^2}\right] \le \frac{8}{(A/\tau)^2}\left[\underbrace{\frac{(L-\frac{1}{2})^4}{5}\sigma^2}_{\text{Noise induced}} + \underbrace{4L^4(L+1)^2(A/4\tau)^{2L}}_{\text{Modeling error}}\right]. \tag{24}$$

Since γ defines a typical scale for the Hamiltonian coefficients, $\sqrt{\mathbb{E}\left[(c_1-\tilde{c}_1)^2/\gamma^2\right]}$ can be interpreted as a relative error.

Proof. We use the identity $\mathbb{E}[(c_1-\tilde{c}_1)^2] = (c_1-\mathbb{E}[c_1])^2 + \mathrm{var}[\tilde{c}_1]$, where $\mathrm{var}[\tilde{c}_1]$ is variance in the estimator $\tilde{c}_1$ due to randomness in the dataset.

$$\mathrm{var}[\tilde{c}_1] = \mathrm{var}\left[\frac{2}{A}\sum_{m=1}^{L-1}(-1)^m\tilde{b}_m m^2\right]$$

Applying Equation (7):

$$= \mathrm{var}\left[\frac{2}{A}\frac{2}{L}\sum_{\ell=1}^{L}y_\ell\sum_{m=1}^{L-1}(-1)^m m^2 T_m(z_\ell)\right]$$

Since each of the y_ℓ are statistically independent:

$$\begin{aligned}
&= \frac{16}{L^2A^2}\sum_{\ell=1}^{L}\sigma_\ell^2\left(\sum_{m=1}^{L-1}(-1)^m m^2 T_m(z_\ell)\right)^2 &(25)\\
&\le \frac{16\sigma^2}{L^2A^2}\sum_{\ell=1}^{L}\left(\sum_{m=1}^{L-1}(-1)^m m^2 T_m(z_\ell)\right)^2 \quad \text{where } \sigma^2 = \max_\ell \sigma_\ell^2\\
&= \frac{16\sigma^2}{L^2A^2}\sum_{m_1=1}^{L-1}\sum_{m_2=1}^{L-1}(-1)^{m_1+m_2}(m_1m_2)^2\sum_{\ell=1}^{L}T_{m_1}(z_\ell)T_{m_2}(z_\ell)
\end{aligned}$$

By the discrete orthogonality conditions, the sum is non-vanishing only when $m_1 = m_2$.

$$\begin{aligned}
&= \frac{16\sigma^2}{L^2A^2}\frac{L}{2}\sum_{m=1}^{L-1}m^4\\
&= \frac{4(L-1)(2L-1)(3L^2-3L-1)}{15A^2}\sigma^2\\
&\le \frac{8\left(L-\frac{1}{2}\right)^4}{5A^2}\sigma^2.
\end{aligned}$$

Next, we evaluate the bias $(c_1-\mathbb{E}[\tilde{c}_1])^2$. Since $\mathbb{E}[\tilde{c}_1]$ corresponds to Chebyshev interpolation with no noise, we make use of a theorem [6, Equation 4.2] concerning the derivative error bounds for Chebyshev interpolation. This theorem says that if $\tilde{f}$ is a degree $L-1$ Chebyshev interpolation of some function f, $\left|f'(0)-\tilde{f}'(0)\right| \le \frac{|\omega_1(0)|\left|f^{(L)}\right|}{L!}$, where $f^{(L)}$ is the Lth derivative of f, $\omega_1(t) \equiv \prod_{\ell=1}^{L-1}(t-\eta_\ell)$, $t_\ell \le \eta_\ell \le t_{\ell+1}$ and $|f| \equiv \sup_{0\le t\le A}|f(t)|$. Applied to our case, $f = \langle P(t)\rangle$, so $f^{(L)}(t) = i^m\,\mathrm{Tr}([H^mP]\rho_0(t)) \implies \left\|f^{(L)}(t)\right\| \le \|[H^mP]\|$. Then:

$$\begin{aligned}
|c_1-\mathbb{E}[\tilde{c}_1]| &\le \left|f'-\tilde{f}'\right|\\
&\le \frac{|\omega_1|}{L!}\left|f^{(L)}\right| &(26)
\end{aligned}$$

Applying Lemma 4:

$$\leq \frac{|\omega_1|}{L!}\gamma^L(L+1)!$$

It remains to upper bound $|\omega_1|$.

$$\begin{aligned}|\omega_1| &\leq \left|\prod_{\ell=1}^{L-1}\frac{A}{2}(-1-z_{\ell+1})\right| \\ &= (A/2)^{L-1}\prod_{\ell=2}^{L}(-1-z_\ell)\end{aligned}$$

Note that $\prod_{\ell=2}^{L}(z-z_\ell) = \frac{T_L(z)}{2^{L-1}(z-z_1)}$, since this is the unique monic polynomial with roots at $z_2,\ldots,z_L$. Then:

$$= (A/2)^{L-1}\left|\frac{\cos(L\arccos(1))}{2^{L-1}(-1-z_1)}\right|$$

We use the fact that $\cos x < 1 - x^2/2.3$ for $|x| < 1$, so that $\left|\frac{1}{-1-z_1}\right| = \frac{1}{1-\cos(\pi/2L)} \leq \frac{1}{(\pi/2L)^2/2.3} \leq L^2$ for $L \geq 2$, so:

$$\leq L^2(A/4)^{L-1}$$

Therefore,

$$|c_1 - \mathbb{E}[\tilde{c}_1]| \leq \frac{4L^2(L+1)(A\gamma/4)^L}{A}$$

In summary, the total error is:

$$\mathbb{E}\left[\frac{(c_1-\tilde{c}_1)^2}{\gamma^2}\right] \leq \frac{1}{(A\gamma)^2}\left[\frac{8(L-\frac{1}{2})^4}{5}\sigma^2 + 16L^4(L+1)^2(A\gamma/4)^{2L}\right].$$

Remark 4.1. The above error bound can be written as:

$$\mathbb{E}\left[\frac{(c_1-\tilde{c}_1)^2}{\gamma^2}\right] \leq \mathcal{O}\left(\frac{\boldsymbol{L^4}}{(A/\tau)^2}\left[\sigma^2 + \boldsymbol{L^2(A/\tau)^{2L}}\right]\right). \tag{27}$$

By viewing the estimator $\tilde{c}_1$ as a generalized finite-difference estimator $f'(0) \approx \frac{f(\epsilon)-f(0)}{\epsilon}$, we argue that the terms in this expression (with the exception of those in bold) are fundamental:

- The inverse dependence on evolution time A corresponds to dependence on $1/(\Delta t)^2$ in the finite-difference estimator.
- The σ^2 dependence originates in the noisiness of measurements we take.

On the other hand, the terms in bold are not fundamental. More precisely, they originate in the fact that we can only evolve our system forward in time. If it were possible to evolve backward in time, we would have access to the central difference estimator $f'(0) \approx \frac{f(\epsilon)-f(-\epsilon)}{2\epsilon}$. This would improve our estimator by reducing the terms in bold.

- Currently, the dependence on $(L+1)(A/\tau)^L$ measures the modeling error. This is analogous to the finite difference estimator $f'(x) \approx \frac{f(x+\epsilon)-f(x)}{\epsilon}$ having an error that scales with $\mathcal{O}\left(\epsilon\frac{f''(\xi)}{2!}\right)$, where $x \leq \xi \leq x+\epsilon$. However, with a central difference method has an error that scales with error $\mathcal{O}\left(\epsilon^2\frac{f'''(\xi)}{3!}\right)$. Therefore, roughly speaking, we would expect the modeling error to improve by at least a factor $\frac{A/\tau}{L+1}$ if we had access to backwards time evolution.

- The L^4 dependence changes to a L^2 dependence. This is because, by placing $t = 0$ in the center of the Chebyshev roots $\{z_i\}$ (rather than at the extreme end $z = -1$), the expression for c_1 changes its form from $\sim \sum_m b_m m^2$ to $\sim \sum_m b_m m$. Plugging this into the derivation for the variance error, we get scaling with L^2.

Theorem 5 (Sample complexity for one coefficient). Fix some maximum failure probability δ and an error ϵ. Assume that we have access to an unbiased (single-shot) estimator of $\langle P(t)\rangle$ with variance $\sigma^2 \le 1$. Furthermore, assume $\|P\| \le 1$. Let $L = \Theta(\log \epsilon^{-1})$, and let L_1 be any positive constant such that $\lim_{\epsilon\to 0^+} \frac{L}{\log \epsilon^{-1}} < L_1$. Let $A = 4e^{-3/L_1}/\gamma = \Theta(\tau)$. Then, with

$$N = \mathcal{O}\big(\log(1/\delta)\,\mathrm{polylog}(1/\epsilon)\epsilon^{-2}\big) \tag{28}$$

sample complexity, we can construct an estimator $\tilde{c}_1$ such that $\frac{|c_1-\tilde{c}_1|}{\gamma} \le \epsilon$, except with failure probability at most δ.

Proof. To ensure $\frac{|c_1-\tilde{c}_1|}{\gamma} \le \epsilon$, it suffices to guarantee $\frac{|\tilde{c}_1-\mathbb{E}[\tilde{c}_1]|}{\gamma} + \frac{|\mathbb{E}[\tilde{c}_1]-c_1|}{\gamma} \le \epsilon$. Since the bias $|\mathbb{E}[\tilde{c}_1] - c_1|$ was shown to be upper bounded by $\frac{4L^2(L+1)(A\gamma/4)^L}{A}$, we require:

$$\frac{|\tilde{c}_1 - \mathbb{E}[\tilde{c}_1]|}{\gamma} \le \epsilon - \frac{4L^2(L+1)(A\gamma/4)^L}{(A\gamma)}$$

with failure probability at most δ. We demonstrate that a sample mean with

$$N = \mathcal{O}\big(\mathrm{polylog}(1/\epsilon)\log(1/\delta)\epsilon^{-2}\big) \tag{29}$$

satisfies this. A simple application of Hoeffding's inequality says that it suffices to have

$$N \le \frac{(c_{max} - c_{min})^2}{2\gamma^2}\log(2/\delta)\left(\epsilon - \frac{4L^2(L+1)(A\gamma/4)^L}{A\gamma}\right)^{-2},$$

where c_{max} and c_{min} are the maximum and minimum possible values for a single-shot estimator of $\tilde{c}_1$. It is simple to extend Equation (25), using the fact that $y_\ell \in [-1, 1]$, to show that $(c_{max} - c_{min})^2 \le \frac{32(L-\frac{1}{2})^4}{5A^2}$. Thus, the upper bound on N becomes:

$$N \le \frac{16}{5}\log(2/\delta)\left(\frac{A\gamma}{L^2}\epsilon - 4(L+1)(A\gamma/4)^L\right)^{-2} \tag{30}$$

Let us choose $L = \Theta(\log \epsilon^{-1})$. That is, there exists some $0 < L_0 < L_1$ such that $L_0 < \lim_{\epsilon\to 0^+} \frac{L}{\log \epsilon^{-1}} < L_1$. Then $(A\gamma/4)^L = \mathcal{O}\big(\epsilon^{L_1 \log(4/A\gamma)}\big)$. If we set $A = 4e^{-3/L_1}/\gamma$, we have $(A\gamma/4)^L = \mathcal{O}\big(\epsilon^3\big)$. Then:

$$= \mathcal{O}\left(\left(\frac{e^{-3L_1}}{\big(L_1 \log \epsilon^{-1}\big)^2}\epsilon - L_1\big(\log \epsilon^{-1}\big)\epsilon^3\right)^{-2}\log(1/\delta)\right) \tag{31}$$

$$= \mathcal{O}\left(\mathrm{polylog}(\epsilon^{-1})\Big(\epsilon - \big(\epsilon \log \epsilon^{-1}\big)^3\Big)^{-2}\log(1/\delta)\right) \tag{32}$$

Since $\log \epsilon^{-1} = \mathcal{O}(\epsilon^{-m})$ for every positive exponent m:

$$= \mathcal{O}\big(\mathrm{polylog}(\epsilon^{-1})\log(1/\delta)\epsilon^{-2}\big) \tag{33}$$

The sample complexity is NL, since L queries are required for a single-shot estimate of $\tilde{c}_1$ (from evaluating the expectation value of the observable at L different evolution times). However, since $L = \Theta(\log \epsilon^{-1})$, we still have $NL = \mathcal{O}\big(\log(1/\delta)\,\mathrm{polylog}(1/\epsilon)\epsilon^{-2}\big)$.

Supplementary Note 4 – Proof of Theorem 1

The following is a sequence of results used when efficiently parallelizing our measurements for Hamiltonian learning with unitary dynamics.

Lemma 6 (Term selection). Let P be some Pauli operator such that there exists some $i \in \{1, \ldots, r\}$ where $\operatorname{supp} P \subseteq \operatorname{supp} P_i$ and $\frac{i[P_i,P]}{2} \neq 0$. Let $\mathcal{X} = \operatorname{supp} P_i$. Let

$$\mathcal{X} = \operatorname{supp} P_i, \tag{34}$$

$$\mathcal{Y} = \left(\bigcup \{\operatorname{supp} P_j \mid \operatorname{supp} P_j \cap \mathcal{X} \neq \varnothing\} \right) \setminus \mathcal{X}, \tag{35}$$

$$\mathcal{Z} = (\mathcal{X} \cup \mathcal{Y})', \tag{36}$$

$$\rho_0 = \left(\frac{\mathbb{I} + i[P_i, P]/2}{2^{|\mathcal{X}|}} \right)^{(\mathcal{X})} \otimes \left(\frac{\mathbb{I}}{2^{|\mathcal{Y}|}} \right)^{(\mathcal{Y})} \otimes \rho_0^{(\mathcal{Z})}. \tag{37}$$

In words, $\mathcal{Y}$ is a neighborhood around $\mathcal{X}$ that contains the support of all Paulis that intersect with $\mathcal{X}$, and $\mathcal{Z}$ is the set of all qubits that are not in $\mathcal{X} \cup \mathcal{Y}$. The state ρ_0 is defined such that for all qubits in $\mathcal{Y}$, it is the maximally mixed state and for qubits inside $\mathcal{X}$, ρ_0 is defined in a way such that $\operatorname{Tr}\left(i[P_i, P]\rho_0^{(\mathcal{X})}/2\right) = 1$, and for all other qubits, ρ_0 can be anything. Then:

$$\operatorname{Tr}(i[H, P]\rho_0/2) = \theta_i. \tag{38}$$

Proof. It suffices to show that for all j such that $j \neq i$, $\operatorname{Tr}(i[P_j, P]\rho_0) = 0$. This is trivially true in the case where $\operatorname{supp} P_j \cap \operatorname{supp} P = \varnothing$, since the commutator vanishes. There are two remaining cases.

CASE 1. P_j acts nontrivially on some set of qubits in $\mathcal{Y}$. Note that $P^{(\mathcal{Y})} = \mathbb{I}$. Then $[P_j, P] = \left[P_j^{(\mathcal{X})}, P^{(\mathcal{X})}\right] \otimes P_j^{(\mathcal{Y})} \otimes \mathbb{I}^{(\mathcal{Z})}$, and:

$$\operatorname{Tr}(i[P_j, P]\rho_0) = \operatorname{Tr}\left(i\left[P_j^{(\mathcal{X})}, P^{(\mathcal{X})}\right]\rho_0^{(\mathcal{X})}\right) \operatorname{Tr}\left(P_j^{(\mathcal{Y})}\rho_0^{(\mathcal{Y})}\right) \operatorname{Tr}\left(\mathbb{I}^{(\mathcal{Z})}\rho_0^{(\mathcal{Z})}\right)$$

Since we assumed $P_j^{(\mathcal{Y})}$ acts nontrivially on at least one qubit in $\mathcal{Y}$, and $\rho_0^{(\mathcal{Y})} \propto \mathbb{I}$, $\operatorname{Tr}\left(P_j^{(\mathcal{Y})}\rho_0^{(\mathcal{Y})}\right) = 0$.

$$= 0$$

CASE 2. P_j acts trivially on all qubits in $\mathcal{Y}$. Then, we have $i[P_j, P] = i\left[P_j^{(\mathcal{X})}, P^{(\mathcal{X})}\right] \otimes \mathbb{I}^{(\mathcal{Y})} \otimes \mathbb{I}^{(\mathcal{Z})}$.

$$\operatorname{Tr}(i[P_j, P]\rho_0) = \operatorname{Tr}\left(i\left[P_j^{(\mathcal{X})}, P^{(\mathcal{X})}\right] \frac{\mathbb{I} + i[P_i, P]/2}{2^{|\mathcal{X}|}}\right)$$

Since $\left[P_j^{(\mathcal{X})}, P^{(\mathcal{X})}\right]$ is traceless:

$$= \frac{\operatorname{Tr}\left(i\left[P_j^{(\mathcal{X})}, P^{(\mathcal{X})}\right] i\left[P_i^{(\mathcal{X})}, P^{(\mathcal{X})}\right]/2\right)}{2^{|\mathcal{X}|}}$$

We now observe that $P_i^{(\mathcal{X})} \neq P_j^{(\mathcal{X})} \implies \left[P_i^{(\mathcal{X})}, P^{(\mathcal{X})}\right] \neq \left[P_j^{(\mathcal{X})}, P^{(\mathcal{X})}\right]$. Since $\left[P_i^{(\mathcal{X})}, P^{(\mathcal{X})}\right]$ and $\left[P_j^{(\mathcal{X})}, P^{(\mathcal{X})}\right]$ are both proportional to different Pauli matrices, by the orthonormality of Pauli matrices, the trace vanishes.

$$= 0$$

Finally:

$$\begin{aligned} \operatorname{Tr}(i[H, P]\rho_0) &= \sum_{j=1}^{n} \theta_j \operatorname{Tr}(i[P_j, P]\rho_0) \\ &= \theta_i \operatorname{Tr}(i[P_i, P]\rho_0) \\ &= 2\theta_i \quad \text{by definition of } \rho_0 \end{aligned}$$

Lemma 7 (Simultaneous inference for a partition). Let $\mathbf{V}_i$ be a partition in a coloring of $\mathcal{G}^2$. The coefficient for each Pauli in $\mathbf{V}_i$ can be inferred with up to an error $\epsilon\|\Theta\|_\infty$, with failure probability for each individual coefficient being at most δ (so the overall failure probability is upper bounded by $\delta|\mathbf{V}_i|$). This can be done with sample complexity

$$\mathcal{O}\big(\mathscr{D}^2\log(1/\delta)\,\mathrm{polylog}(\mathscr{D}/\epsilon)\epsilon^{-2}\big). \tag{39}$$

Proof. For each $P_{i,j}\in\mathbf{V}_i$, let $P'_{i,j}$ be a single-qubit Pauli such that $\big[P_{i,j},P'_{i,j}\big]\neq 0$. Let $M=(\mathrm{supp}\,\mathbf{V}_i)'$. Let

$$\rho_0=\left(\bigotimes_{j;P_{i,j}\in\mathbf{V}_i}\left(\frac{\mathbb{I}+P'_{i,j}}{2}\right)\right)\otimes\left(\frac{\mathbb{I}}{2^{|M|}}\right)^{(M)} \tag{40}$$

Here, M is the "moat". Now, for any $P_{i,j}$, there is a straightforward labeling of qubits that maps ρ_0 onto the structure $\rho_0^{(\mathcal{X})}\otimes\rho_0^{(\mathcal{Y})}\otimes\rho_0^{(\mathcal{Z})}$ in Lemma 6.

$$\mathcal{X}=\mathrm{supp}(P_j) \tag{41}$$

$$\mathcal{Y}=M \tag{42}$$

$$\mathcal{Z}=\mathrm{supp}\,\mathbf{V}_i\setminus\mathrm{supp}\,P_{i,j} \tag{43}$$

By Lemma 6, so long as we are able to evaluate expectation values $\mathrm{Tr}\big(P'_{i,j}\rho_0(t)\big)$, we can find $\mathrm{Tr}\big(i\big[H,P'_{i,j}\big]\rho_0\big)$, hence we can also find $\{\theta_{i,j}\mid P_{i,j}\in\mathbf{V}_i\}$. Therefore, it suffices to show that (for some fixed time t) the expectation values $\mathrm{Tr}\big(P'_{i,j}\rho_0(t)\big)$ can be evaluated for all j. This is not difficult, since $\{P'_{i,j}\}$ is a set of Pauli operators with non-overlapping support, so they commute and can be measured simultaneously.

Since the error in Theorem 5 was relative to $\gamma\equiv 2\mathscr{D}\|\Theta\|_\infty$, after rescaling $\epsilon\to\frac{\epsilon}{2\mathscr{D}}$ we find that we require $L\sim\log\epsilon^{-1}$ groups of $N\sim\mathscr{D}^2\,\mathrm{polylog}(\mathscr{D}/\epsilon)\log(1/\delta)\epsilon^{-2}$ measurements. The total sample complexity is NL, which gives the complexity in Equation (39).

Finally, since our parallelization technique relies on a graph coloring, we provide a formal definition below.

Definition 3 (Graph coloring). A C-coloring of a graph is a labelling of the vertices in a graph with exactly C colors such that no two vertices with the same color share an edge. The minimum number of colors required to color a graph $\mathcal{G}$ is known as its chromatic number $\chi(\mathcal{G})$. We will write a C-coloring as $\{\mathbf{V}_i\mid i=1,\ldots,C\}$, where $\mathbf{V}_i=\{P_{i,1},P_{i,2},\ldots\}$ is called a partition, and is the set of Paulis that is colored with i. We will also define the support of a partition to be:

$$\mathrm{supp}\,\mathbf{V}_i=\bigcup_{P_{i,j}\in\mathbf{V}_i}\mathrm{supp}(P_{i,j}). \tag{44}$$

Furthermore, by Vizing's theorem [7], any graph with degree Δ can be colored with at most $\chi(\mathcal{G})\leq\Delta+1$ colors. This coloring can be found in $\mathcal{O}(\Delta)$ time with a greedy algorithm [8]. Since the squared interaction graph has degree at most at most $\mathscr{D}(\mathscr{D}-1)+1=\mathscr{D}^2-\mathscr{D}+1$, the graph can be colored with at most $\mathscr{D}^2-\mathscr{D}+2$ colors. For non-trivial Hamiltonians ($\mathscr{D}\geq 2$), this is at most $\mathscr{D}^2$ colors.

Example 3.1. The following is an example of a coloring of the squared interaction graph $\mathcal{G}^2$ for the 9-qubit TFIM model.

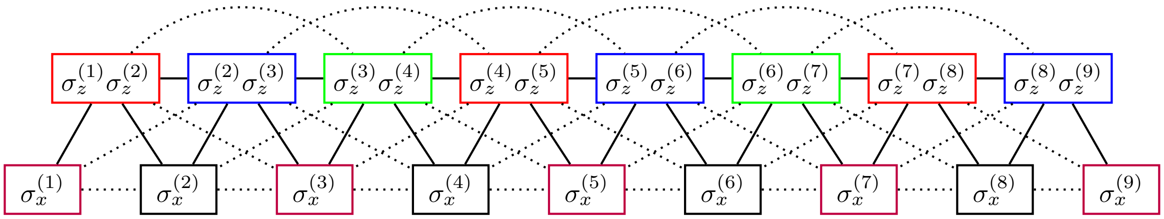

Supplementary Figure 3: A 5-coloring of the squared interaction graph $\mathcal{G}^2$. Solid edges indicate the distance between the nodes is 1 in $\mathcal{G}$, and dashed edges indicate the distance in $\mathcal{G}$ is 2.

Theorem 6 (Hamiltonian learning with unitary dynamics). Fix a sparsely interacting Hamiltonian H that has r terms in its Pauli expansion with coefficients Θ. For the appropriate choice of Chebyshev degree L and evolution time A, Algorithm 4 of the main text solves the quantum Hamiltonian learning problem (with an additive error $\epsilon\|\Theta\|_\infty$ and failure probability at most δ) with sample complexity

$$\mathcal{O}\left(\frac{\mathscr{D}^4 \log(r/\delta)\,\mathrm{polylog}(\mathscr{D}/\epsilon)}{\epsilon^2}\right), \tag{45}$$

and classical processing time complexity

$$\mathcal{O}\left(\frac{\mathscr{D}^2 r \log(r/\delta)\,\mathrm{polylog}(\mathscr{D}/\epsilon)}{\epsilon^2}\right). \tag{46}$$

Proof. After finding a $\mathscr{D}^2$ coloring for the squared interaction graph $\mathcal{G}^2$, we use the result from Lemma 7 to simultaneously infer the coefficients for each partition in the graph coloring. For each partition $\mathbf{V}_i$, with reference to Theorem 5, it suffices to set $N = \mathcal{O}\big(\mathrm{polylog}(\mathscr{D}/\epsilon)(\epsilon/\mathscr{D})^{-2}\big)$ and $L = \mathcal{O}\big(\log\big(\epsilon^{-1}\big)\big)$ to ensure that each individual coefficient can be recovered up to $|\theta_{true} - \theta_{infer}| \leq \epsilon\|\Theta\|_\infty$ with failure probability at most $\frac{\delta}{r}$. By doing this for each of the $\chi(\mathcal{G}^2) \leq \mathscr{D}^2 + 1$ partitions and applying a union bound on the failure probability, we see we can recover each coefficient up to an additive error $\epsilon\|\Theta\|_\infty$ with failure probability at most δ using $N \cdot L \cdot \chi(\mathcal{G}^2)$ queries, which has a complexity given by Equation (45). The classical time complexity takes a similar form to the sample complexity, except it replaces a factor of $\mathscr{D}^2$ (corresponding to $\chi(\mathcal{G}^2)$) by a factor r. This is because we need to process NL measurement results for each of the r coefficients in the Hamiltonian, whereas for the sample complexity, we make NL measurements for each of the $\chi(\mathcal{G}^2)$ partitions. Since the classical time to color the graph is just $\mathcal{O}\big(\mathscr{D}^2\big)$, the overall classical complexity is still dominated by $N \cdot L \cdot r$, giving Equation (46).

Supplementary Note 5 – Hamiltonian Learning with Gibbs States

Lemma 8. If P_i is a term in the Hamiltonian, the expectation $\langle P_i \rangle_\beta$ can also be written as:

$$\langle P_i \rangle_\beta = -\frac{1}{\beta}\frac{\partial}{\partial\theta_i}\log\operatorname{Tr}\exp(-\beta H) \tag{47}$$

Proof. This is Proposition 3.2 of Haah *et al.* [3]. We reproduce the proof here for completeness:

$$\begin{aligned}
-\frac{1}{\beta}\frac{\partial}{\partial\theta_i}\log\operatorname{Tr}\exp(-\beta H) &= -\frac{1}{\beta}\frac{\operatorname{Tr}\frac{\partial}{\partial\theta_i}\exp(-\beta H)}{\operatorname{Tr}\exp(-\beta H)}\\
&= -\frac{1}{\beta}\frac{\operatorname{Tr}(-\beta P_i\exp(-\beta H))}{\operatorname{Tr}\exp(-\beta H)}\\
&= \frac{\operatorname{Tr}(P_i\exp(-\beta H))}{\operatorname{Tr}\exp(-\beta H)}.
\end{aligned}$$

We define $\mathcal{L} = \log\operatorname{Tr}\exp(-\beta H)$ and view this as a function of $\beta, \theta_1, \ldots, \theta_r$.

Lemma 9. Using a multivariate Taylor expansion, we can write

$$\mathcal{L} = \sum_{m\geq 0}\beta^m\sum_{\alpha;|\alpha|=m}\frac{\Theta^\alpha}{\alpha!}\mathcal{D}_\alpha\mathcal{L}, \tag{48}$$

where α are the multi-index sets defined in Definition 1. The derivative operator $\mathcal{D}_\alpha = \frac{\partial}{\partial z_1^{\alpha_1}\ldots\partial z_r^{\alpha_r}}$ is evaluated at $\Theta = 0$. We have defined $z_i \equiv \beta\theta_i$. Furthermore:

$$\langle P_i \rangle_\beta = -\sum_{m\geq 0}\beta^m\sum_{\alpha;|\alpha|=m}\frac{\Theta^\alpha}{\alpha!}\mathcal{D}_{\alpha'}\mathcal{L} \tag{49}$$

where $\alpha' = (\alpha_1, \ldots, \alpha_i + 1, \ldots, \alpha_r)$.

Proof. The first statement follows directly from Equation (22) of Haah *et al.* [3]. The second statement follows because the derivatives $\frac{\partial}{\partial z_i}$ and $\frac{\partial}{\partial z_j}$ commute for all i, j, so the operator $-\frac{1}{\beta}\frac{\partial}{\partial\theta_i} = -\frac{\partial}{\partial z_i}$ from Equation (47) can be distributed into the sum in Equation (48).

Remark 6.1. $\mathcal{D}_\alpha \mathcal{L}$ is a constant that does not depend on β. To see this, observe that we can write $\mathcal{L} = \log \operatorname{Tr} \exp(-\sum_i z_i P_i)$ as a function of z_i alone. Therefore, evaluating the derivative at $z_1 = \ldots = z_r = 0$ with the operator $\mathcal{D}_\alpha$ yields a constant independent of β. So, we are shifting our viewpoint of $\langle P_i \rangle_\beta$ as a polynomial in β with coefficients $\sum_{\alpha;|\alpha|=m} \frac{\Theta^\alpha}{\alpha!} \mathcal{D}_{\alpha'} \mathcal{L}$, rather than as a multivariate polynomial in the coefficients Θ (as done in Haah *et al.* [3]).

Remark 6.2. The zeroth order term in $\langle P_i \rangle_\beta$ must vanish, since $\langle P_i \rangle_{\beta=0}$ corresponds to evaluating P_i on the maximally mixed state. The first order term is can be found by differentiating Equation (47) and evaluating at $\beta = 0$:

$$
\begin{aligned}
\frac{\mathrm{d}}{\mathrm{d}\beta} \langle P_i \rangle_{\beta=0} &= \frac{\operatorname{Tr}(\exp(-\beta H)) \frac{\mathrm{d}}{\mathrm{d}\beta} \operatorname{Tr}(P_i \exp(-\beta H)) - \operatorname{Tr}(P_i \exp(-\beta H)) \frac{\mathrm{d}}{\mathrm{d}\beta} \operatorname{Tr}(\exp(-\beta H))}{\operatorname{Tr}(\exp(-\beta H))^2} \\
&= \frac{1}{2^n} \frac{\mathrm{d}}{\mathrm{d}\beta} \operatorname{Tr}(P_i \exp(-\beta H)) \quad \text{where } n \text{ is the number of qubits} \\
&= \frac{1}{2^n} \operatorname{Tr}(-P_i H \exp(-\beta H)) \\
&= \frac{1}{2^n} \operatorname{Tr}(-P_i H) \\
&= -\theta_i \quad \text{by the orthogonality of the Pauli matrices}
\end{aligned} \tag{50}
$$

Lemma 10 (Temperature derivative bound). The mth derivative of $\langle P_i \rangle_\beta$ evaluated at $\beta = 0$ is bounded in absolute value by:

$$
\left| \frac{\mathrm{d}^m \langle P_i \rangle_\beta}{\mathrm{d}\beta^m} \right|_{\beta=0} \leq \|\Theta\|_\infty^m (m+1)! (2e^2(\mathscr{D}^2 - 1))^{m+1} \tag{51}
$$

Proof. This follows directly from Lemma 3.7 and Proposition 3.8 of Haah *et al.* [3]. First, we apply Proposition 3.8 to find that $\left| \frac{\mathcal{D}_{\alpha'} \mathcal{L}}{\alpha!} \right| \leq m \left| \frac{\mathcal{D}_{\alpha'} \mathcal{L}}{\alpha'!} \right| \leq m(2e(\mathscr{D}+1))^{m+1}$. Next, we observe that $\mathcal{D}_{\alpha'} \mathcal{L}$ is vanishing if α' does not induce a connected subgraph of the Hamiltonian interaction graph. By Lemma 3.7, there are at most $e\mathscr{D}(e(\mathscr{D}-1))^m$ such α'. Therefore:

$$
\begin{aligned}
\left| \frac{\mathrm{d}^m \langle P_i \rangle_\beta}{\mathrm{d}\beta^m} \right|_{\beta=0} &= m! \left| \sum_{\alpha;|\alpha|=m} \frac{\Theta^\alpha}{\alpha!} \mathcal{D}_{\alpha'} \mathcal{L} \right| \\
&\leq \|\Theta\|_\infty^m (m+1)! e\mathscr{D}(e(\mathscr{D}-1))^m (2e(\mathscr{D}+1))^{m+1} \\
&\leq \|\Theta\|_\infty^m (m+1)! (2e^2(\mathscr{D}^2 - 1))^{m+1}
\end{aligned}
$$

With unitary dynamics, we already demonstrated that the derivative bound drops by a factor $m!$ if the Hamiltonian is commuting. We expect a similar decrease here, but we leave a proof of this for future works.

Theorem 7 (Hamiltonian learning with Gibbs states). The Hamiltonian learning problem (with an additive error $\epsilon\|\Theta\|_\infty$ and failure probability at most δ) can be solved using

$$
\mathcal{O}\left(\frac{\mathscr{D}^5 \log(r/\delta) \operatorname{polylog}(\mathscr{D}/\epsilon)}{\epsilon^2} \right) \tag{52}
$$

copies of the Gibbs state. This can be achieved with a time complexity

$$
\mathcal{O}\left(\frac{\mathscr{D}^4 r \log(1/\delta) \operatorname{polylog}(\mathscr{D}/\epsilon)}{\epsilon^2} \right). \tag{53}
$$

Proof. The protocol is a near mirror image of the Hamiltonian learning protocol using unitary dynamics. We aim to infer the first derivative in the polynomial $\langle P_i \rangle_\beta$, so we apply our EstimateDerivative protocol from Algorithm 2. The error scaling of this protocol is slightly different, since $\|f^{(L)}\|$ changes using $\langle P_i \rangle_\beta$ as the polynomial rather than $\langle P_i(t) \rangle$. Repeating the analysis from Equation (26), we find:

$$
\begin{aligned}
|c_1 - \mathbb{E}[\tilde{c}_1]| &\leq \frac{L^2 (A/4)^{L-1}}{L!} \left| \frac{\mathrm{d}^m \langle P_i \rangle_\beta}{\mathrm{d}\beta^m} \right|_{\beta=0} \\
&\leq \|\Theta\|_\infty^L L^2 (L+1)(A/4)^{L-1} (2e^2(\mathscr{D}^2 - 1))^{L+1} \\
&= \mathcal{O}\big(L^3 (A\|\Theta\|_\infty \mathscr{D}^2)^L\big)
\end{aligned}
$$

This amounts to redefining $\gamma = \mathcal{O}\big(\|\Theta\|_\infty \mathscr{D}^2\big)$. This can be plugged into our analysis for Theorem 5 to find that we can infer any individual coefficient up to an error $\epsilon\|\Theta\|_\infty$ with maximum failure probability δ using

$$\mathcal{O}\big(\log(1/\delta)\,\mathrm{polylog}(\mathscr{D}/\epsilon)(\epsilon/\mathscr{D}^2)^{-2}\big) \tag{54}$$

copies of the Gibbs state.

This then carries into our main result Theorem 6. We color our interaction graph such that terms from the same color (i.e., partition) do not have an overlapping support. Then, the coefficients for each partition can be inferred simultaneously because the observables in each partition can be measured simultaneously. Since there are at most $\mathscr{D}+1$ partitions, the overall sample complexity of our algorithm is:

$$\mathcal{O}\big(\mathscr{D}^5\log(1/\delta)\,\mathrm{polylog}(\mathscr{D}/\epsilon)\epsilon^{-2}\big).$$

The processing time is at most

$$\mathcal{O}\big(\mathscr{D}^4 r\log(1/\delta)\,\mathrm{polylog}(\mathscr{D}/\epsilon)\epsilon^{-2}\big),$$

since we need to process $\mathcal{O}\big(\log(1/\delta)\,\mathrm{polylog}(\mathscr{D}/\epsilon)(\epsilon/\mathscr{D}^2)^{-2}\big)$ measurements for each of the r coefficients in the Hamiltonian.

Supplementary Note 6 – Heuristic Optimizations

In the following, we provide a theoretical justification for Optimizations 1 and 2.

Optimization 1. The support tree $\mathcal{T}_P$ is not often a truly regular tree (i.e., not all nodes have exactly $\mathscr{D}+1$ children). We expect this to be reflected in the scaling of the number of non-vanishing tuples. Corollary 2.1 says that the number of non-vanishing tuples of size m is $\mathscr{D}^m(m+1)!$. The factor $\mathscr{D}^m$ resembles the number of nodes at a depth m in a $\mathscr{D}$-regular tree. Therefore, it is reasonable to suppose that we can replace $\mathscr{D}^m$ with a factor that more closely reflects the number of nodes at a depth m in the support tree $\mathcal{T}_P$, which is not a truly regular tree.

We assume $\mathcal{T}_P$ can be modeled as a branching process [9]. A branching process is a stochastic process that models reproduction over m generations. We begin with a population size of $P_0 = 1$, and at each time step, each member of the population produces a random number N of offspring, where N is a positive discrete random variable, and the previous generation dies out. Therefore, the distribution of population sizes is

$$\mathbb{P}(P_{i+1} = x \mid P_i = k) = \mathbb{P}\left(\sum_{j=1}^{k} N_j = x\right). \tag{55}$$

It is a standard result that the expected population size at a time t is $\mathbb{E}[N]^t$ [9]. Applied to our case, we see that a reasonable definition for N is to let it be chosen uniformly at random from the set of degrees in the interaction graph: $N \in_R \{\deg(v) \mid v \in V\}$ (where $\in_R$ denotes random selection). Furthermore, recall Remark 2.1: not every subtree of the support tree $\mathcal{T}_P$ is nonvanishing. For any fixed Pauli, the probability that it commutes with some random Pauli is one-half. This is because two Pauli operators $A = A_1A_2\ldots A_n$ and $B = B_1B_2\ldots B_n$ (with each A_i, B_i being one Pauli matrix) commute when there is an even number of sites where $[A_i, B_i] \neq 0$. Since every single-qubit Pauli matrix does not commute with exactly 2 out of 4 single-qubit Pauli matrices, this yields a one-half probability that A and B commute for B chosen uniformly at random from all n-qubit Pauli matrices. Therefore, as we are constructing a non-vanishing tuple (using the subtree analogy from Corollary 2.1), we expect that adding any node has a one-half probability of commuting with the current subtree. Therefore, we define the average degree to be

$$\overline{\mathscr{D}} = \frac{1}{2|V|}\sum_{v\in V}\deg(v). \tag{56}$$

The factor $\frac{1}{2}$ comes from the fact that for a given subtree, we expect one out of every two of the children that we add to commute with the existing operator defined by the subtree. With this, we now replace $\mathscr{D}$ everywhere with $\overline{\mathscr{D}}$.

Numerical simulations indicate the bound tightening in Optimization 1 is reasonable (Figure 4), and significantly improves algorithm performance (Figure 5). Since the theoretical error bound is linear in $\mathscr{D}$, the improvement in performance is linear in $\mathscr{D}/\overline{\mathscr{D}}$, resulting in a decrease in the number of required queries by a factor $\gtrsim (\mathscr{D}/\overline{\mathscr{D}})^2$.

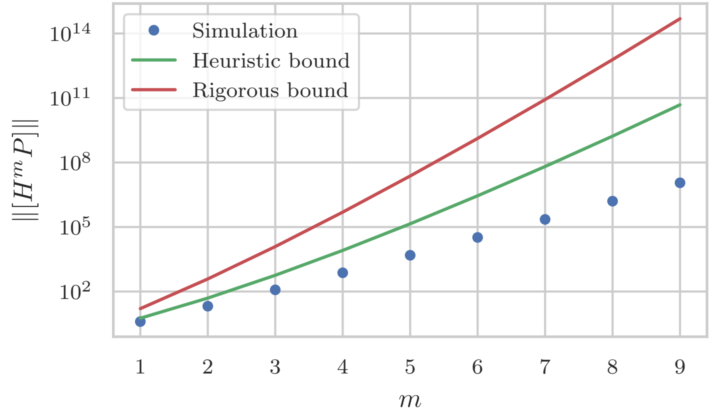

Supplementary Figure 4: Bounds for the iterated commutator norm $[H^m P]$. We randomly generate 10000 TFIM Hamiltonians (sampling the interaction strengths from Unif$(-1, 1)$) for system sizes $n = 2, \ldots, 9$, and calculate the norm for $[H^m P]$ for every single-qubit Pauli P. The points marked simulation in the figure are the maximum $\|[H^m P]\|$ over all 10000 randomly generated Hamiltonians, all single-qubit Paulis, and all system sizes. The rigorous bound is that found in Corollary 2.1: $\mathscr{D}^m(m+1)!$, and the heuristic bound is simply $\overline{\mathscr{D}}^m(m+1)!$. The average degree $\overline{\mathscr{D}}$ was set to 3/2, which is the effective degree as $n \to \infty$.

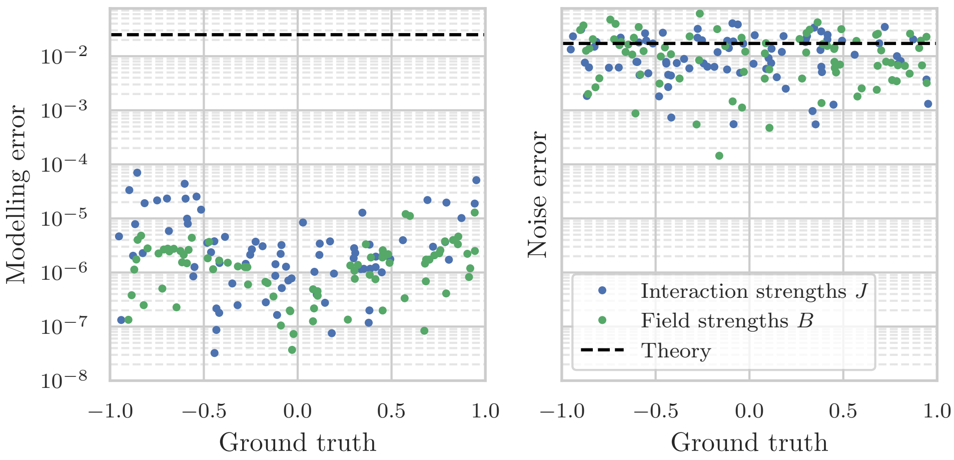

Supplementary Figure 5: The empirical modelling and noise error of the Hamiltonian learning protocol using the optimal A and L. This differs from Figure 5 of the main text in that here we use $\overline{\mathscr{D}}$ rather than $\mathscr{D}$ when calculating the evolution time. We observe a significantly improved agreement between the theoretical modelling error bound and the true modelling error.

Optimization 2. For a fixed number of queries, we can optimize the noise error in Equation (25) by allocating a different number of queries to each of the y_ℓ. If we allocated N_ℓ measurements to each time step, we have $\sigma_\ell^2 \le \frac{1}{N_\ell}$, in which case we solve:

$$\underset{\{\sigma_\ell\}}{\text{minimize}} \left[\frac{16}{L^2 A^2} \sum_{\ell=1}^{L} \sigma_\ell^2 \left(\sum_{m=1}^{L-1} (-1)^m m^2 T_m(z_\ell) \right)^2 \right]$$

$$\text{subject to: } \sum_{\ell=1}^{L} N_\ell \le N_{max}$$

We minimize an upper bound of the objective by replacing σ_ℓ^2 with $\frac{1}{N_\ell}$, in which case the optimization problem can be

solved using the method of Lagrange multipliers, and by introducing a slack variable s so that the constraint becomes $\sum_{\ell=1}^{L} N_\ell - N_{max} - s^2 = 0$. We write $c_\ell \equiv \frac{16}{L^2 A^2}\left(\sum_{m=1}^{L-1}(-1)^m m^2 T_m(z_\ell)\right)^2$ for brevity and obtain:

$$\boldsymbol{\nabla}_{\{N_\ell\},s,\lambda}\left[\sum_{\ell=1}^{L}\frac{c_\ell}{N_\ell} + \lambda\left(\sum_{\ell=1}^{L} N_\ell - N_{max} - s^2\right)\right] = 0$$

This reduces to:

$$\frac{N_{\ell_1}}{N_{\ell_2}} = \frac{\sqrt{c_{\ell_1}}}{\sqrt{c_{\ell_2}}}, \quad \forall \ell_1, \ell_2 = 1, \ldots, L \quad \text{and} \quad \sum_{\ell=1}^{L} N_\ell = N_{max}$$

This yields a closed form expression for N_ℓ:

$$N_\ell = \frac{\sqrt{c_\ell}}{\sum_\ell \sqrt{c_\ell}} N_{max} \tag{57}$$

Optimization 3. Since we know the polynomial at $t = 0$ to have a value of 0 (our SPAM parameters are set such that this is always true), we constrain our solution for the Chebyshev coefficients such that the constant term in the polynomial is 0. That is, rather than fitting a generic polynomial $\sum_{\ell=0} c_\ell t^\ell$ to the data, we fit $\sum_{\boldsymbol{\ell=1}} c_\ell t^\ell$. This further reduces the variance of our solution, mitigating the noise-induced error.

---

[1] J. C. Mason and D. Handscomb, *Chebyshev polynomials* (Chapman & Hall/CRC, 2003).

[2] W. H. Press, ed., *Numerical recipes: the art of scientific computing*, 3rd ed. (Cambridge University Press, Cambridge, UK; New York, 2007).

[3] J. Haah, R. Kothari, and E. Tang, Optimal learning of quantum hamiltonians from high-temperature gibbs states (2021), arXiv:2108.04842 [quant-ph].

[4] N. Alon, Random Structures & Algorithms **2**, 10.1002/rsa.3240020403 (1991).

[5] D. E. Knuth, *The Art of Computer Programming*, Vol. 1 (Addison-Wesley, Reading, Mass, 1969) p. 396, excercise 11.

[6] G. W. Howell, Journal of Approximation Theory **67**, 10.1016/0021-9045(91)90015-3 (1991).

[7] V. G. Vizing, Cybernetics **1**, 10.1007/BF01885700 (1965).

[8] J. Mitchem, The Computer Journal **19**, 182 (1976).

[9] K. B. Athreya and P. E. Ney, *Branching Processes* (Springer Berlin Heidelberg, 1972).
